# Overview of cellular homeostasis-associated nuclear envelope lamins and associated input signals

**DOI:** 10.3389/fcell.2023.1173514

**Published:** 2023-05-12

**Authors:** Hyeong Jae Kim, Peter C. W. Lee, Jeong Hee Hong

**Affiliations:** ^1^ Department of Physiology, Lee Gil Ya Cancer and Diabetes Institute, Gachon University, Incheon, Republic of Korea; ^2^ Lung Cancer Research Center, Asan Medical Center, College of Medicine, University of Ulsan, Seoul, Republic of Korea

**Keywords:** lamin, nuclear envelope, scenescence, redox hemostasis, cell cycle

## Abstract

With the discovery of the role of the nuclear envelope protein lamin in human genetic diseases, further diverse roles of lamins have been elucidated. The roles of lamins have been addressed in cellular homeostasis including gene regulation, cell cycle, cellular senescence, adipogenesis, bone remodeling as well as modulation of cancer biology. Features of laminopathies line with oxidative stress-associated cellular senescence, differentiation, and longevity and share with downstream of aging-oxidative stress. Thus, in this review, we highlighted various roles of lamin as key molecule of nuclear maintenance, specially lamin-A/C, and mutated *LMNA* gene clearly reveal aging-related genetic phenotypes, such as enhanced differentiation, adipogenesis, and osteoporosis. The modulatory roles of lamin-A/C in stem cell differentiation, skin, cardiac regulation, and oncology have also been elucidated. In addition to recent advances in laminopathies, we highlighted for the first kinase-dependent nuclear lamin biology and recently developed modulatory mechanisms or effector signals of lamin regulation. Advanced knowledge of the lamin-A/C proteins as diverse signaling modulators might be biological key to unlocking the complex signaling of aging-related human diseases and homeostasis in cellular process.

## 1 Lamins and laminopathies

**FIGURE 1:**
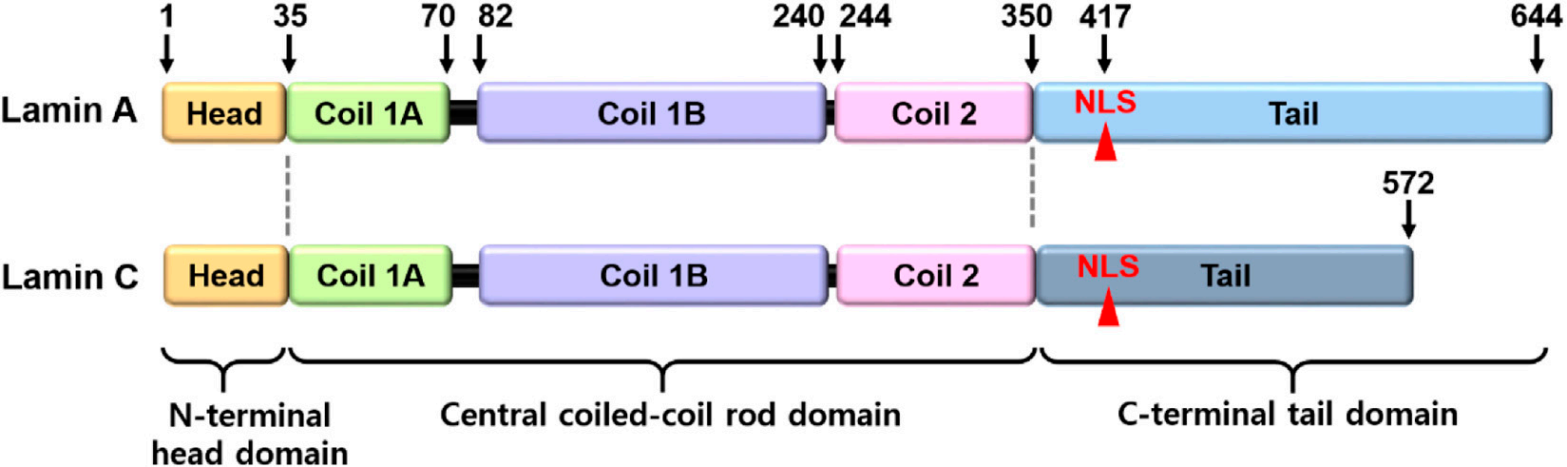
Structural illustration of lamin-A/C. The lamin-A and its isoform C comprise alternative splice variants. NLS, nuclear location signal.

Lamins are the inner nuclear membrane proteins, which associate filament assembly in nuclear proteins to contribute the integrity of nuclear envelope and perform various cellular functions, including fibrous organization of the nuclear envelope, gene regulation, cellular senescence, and differentiation (Aaronson and Blobel, 1975; Aebi et al., 1986; Fisher et al., 1986; Goldman et al., 2002; Dechat et al., 2008). The mammalian lamins generally consist of two major types, A and B, encoded by *LMNA* and *LMNB*, respectively. The cDNA sequences of lamin-A and its isoform C are similar and comprise alternative splice variants that possess partial amino acid sequence identity, whereas the B-type lamins (Lamin B1 and B2) are different (Fisher et al., 1986; Prokocimer et al., 2009). B-type lamins localize in all types of nuclei and are especially involved in brain organogenesis (Evangelisti et al., 2022; Matias et al., 2022), whereas lamin-A proteins are regulated during development (Prokocimer et al., 2009; Vidal et al., 2012) and are involved in the signaling mechanism of various tissues, including liver tissue, hepatocarcinoma cells (Aebi et al., 1986; Hytiroglou et al., 1993), prostate cancer cells (Kong et al., 2012), fibroblasts (Pekovic et al., 2007), osteoblasts (Akter et al., 2009), and bone marrow-derived mesenchymal stem cells (MSCs) (Bermeo et al., 2015). Structurally, lamin-A/C consists of N-terminal head domain, central coiled-coil rod domain, and C-terminal tail domain including nuclear location signal, and CXXF amino acid-specific motif (Figure 1) (Steinle et al., 2004; Ahn et al., 2019). Nuclear lamin proteins localize inner nuclear membrane to provide nuclear structural maintenance through the interaction with nuclear proteins directly or indirectly (Crisp and Burke, 2008; Prokocimer et al., 2009). In addition, lamin proteins bind to DNA and chromatin to maintain nuclear matrix homeostasis (Luderus et al., 1994; Baricheva et al., 1996). Thus, the features of structure-function crosstalk provide mechanical support of nuclear structure and determination of cellular fate.

Since the identification of the lamin-A/C, more than 600 *LMNA* mutations have been discovered in the human genome and mutations of *LMNA* mediate defects in nuclear filament assembly and impaired maturation of lamin A (Ho and Hegele, 2019; Lazarte and Hegele, 2021). Its diversities of mutation have been identified in various human diseases such as Hutchinson–Gilford progeria (HGP) syndrome (Mounkes et al., 2003; Goldman et al., 2004), Emery–Dreifuss muscular dystrophy (EDMD) (Bonne et al., 1999), cardiomyopathy (Ben Yaou et al., 2006; Lu et al., 2011), limb-girdle muscular dystrophy type 1 B (Muchir et al., 2003), Dunnigan-type familial partial lipodystrophy (Merideth et al., 2008), and Charcot–Marie–Tooth syndrome type 2B1 (Mounkes et al., 2003). Various studies have addressed the multiple disordered spectra of lamin mutations, which have terms as laminopathies, based on recent advances.

**FIGURE 2:**
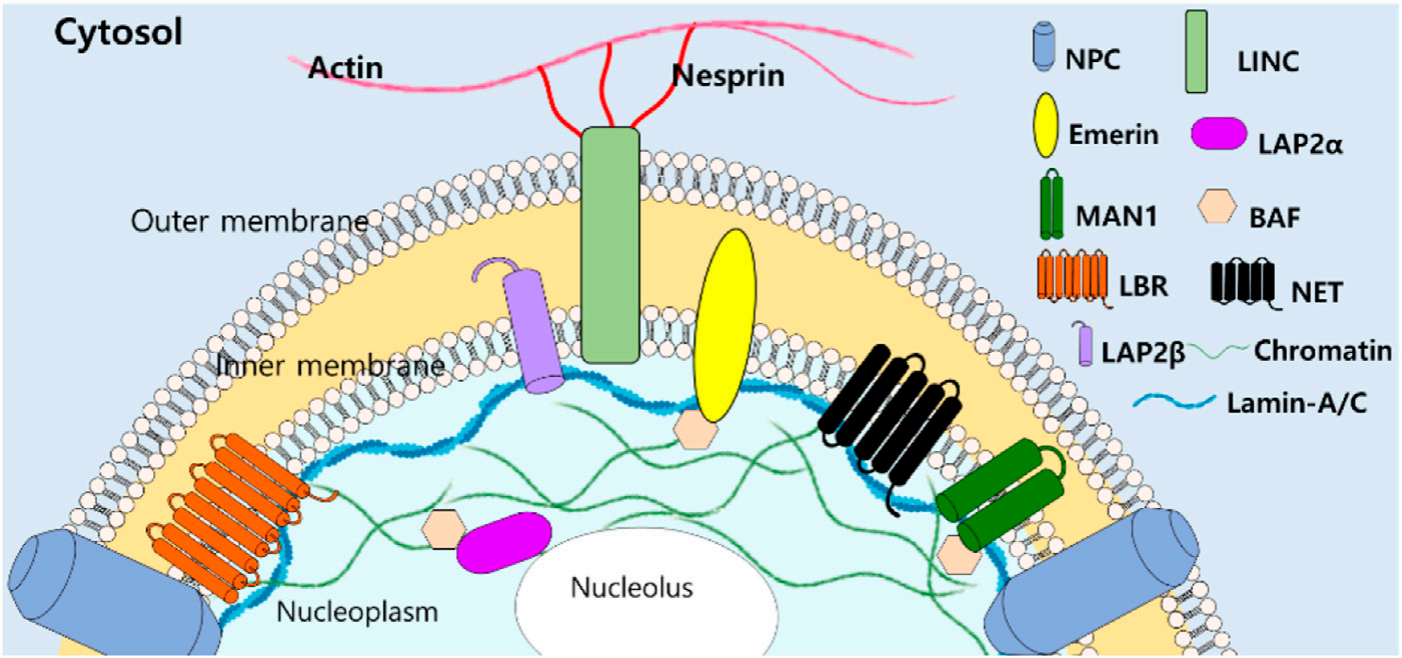
Structural illustration of lamin-A/C-interacting molecules with filamentous structure. NPC, nuclear pore complexes, LINC, linker of nucleoskeleton and cytoskeleton, LEM domain-containing proteins (Emerin, LAP2, and MAN1), BAF: barrier to autointegration factor, LBR: lamin B receptor, NET: nuclear envelope transmembrane proteins (Patil and Sengupta, 2021).

Since lamin-A/C have been addressed as structural “guardians of the soma” (Vidal et al., 2012), various roles of lamins and its modulator signals have been addressed past few decades. Function-associated nuclear structure of lamins and its interacting nuclear molecules (Figure 2) reveal tissue-specific manner and are extensively reviewed (Worman and Schirmer, 2015; Pradhan et al., 2020; Patil and Sengupta, 2021).

**FIGURE 3:**
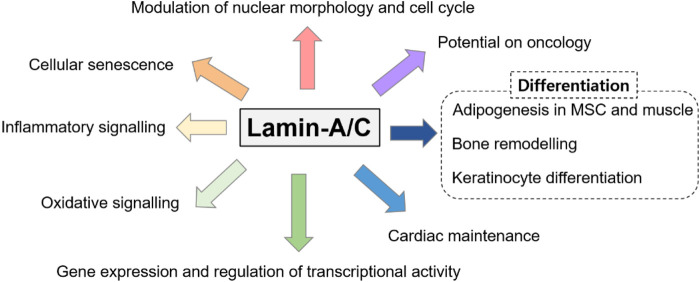
Various roles of lamin-A/C on multiple cellular systems. Various studies have addressed the multiple disordered spectra of lamin mutations and we summarized the diversity of lamin-A/C function.

Genetically, *LMNA* mutations are also multiple and reveal various disturbed cellular functions. Moreover, dysregulated *LMNA* gene is often associated with aging and redox homeostasis-associated features. For example, cells from patients with laminopathies reveal the enhanced reactive oxygen species level (Caron et al., 2007; Malhas and Vaux, 2011; Lattanzi et al., 2012). The phenotypic spectrum of laminopathies is very broad because of the high genetic heterogeneity and phenotypic variability and its features seems to share with downstream of aging-oxidative stress. Thus, we focused various roles of lamin-A/C based on its variability throughout the cellular system and summarized recently identified effector signals on lamin-A/C regulation based on the experimental evidences (Figure 3).

## 2 Cellular roles of lamin-A/C

### 2.1 Modulation of nuclear morphology and cell cycle

It is well known that lamin plays a role in the morphological and mechanical maintenance of the nuclear shape (Gotzmann and Foisner, 2006; Prokocimer et al., 2009; Worman, 2012). Although the roles of lamins are described in chromatin modulation and highlighted in *Drosophila*, *Xenopus*, and *Caenorhabditis elegans*, we summarized mammalian-based or disease-related lamin-A/C research. It has been well determined the role of lamin in the structural maintenance of chromatin modulation, cellular signaling, cell proliferation, and emerging roles in viral infection (Cenni et al., 2008; Dechat et al., 2008; Parnaik, 2008; Prokocimer et al., 2009; Piekarowicz et al., 2017). Maintenance of lamin shows the modulating sign of cellular fate. Proteolytic cleavage of lamin-A enhances the destruction of the nuclear shape and cellular apoptosis (Rao et al., 1996; Takahashi et al., 1996). Non-cleaved mutants of lamin-A or -B induce abnormal chromatin morphology and delayed apoptosis (Rao et al., 1996). A premature aging disorder, the HGP syndrome is caused by a human lamin-A (*LMNA*) mutation and presents aged appearances such as osteoporosis and loss of hair (Eriksson et al., 2003). Cells transfected with 50 amino acid-deleted lamin A and HGP syndrome–derived cells (G608G mutation in the *LMNA* gene) reveal enhanced deformity of the nuclear envelope and chromatin structure (Goldman et al., 2004).

In addition to nuclear maintenance role of lamin, lamin-A/C binds to lamina-associated polypeptide LAP-2α and is anchored to retinoblastoma protein Rb (pRb) (Markiewicz et al., 2002). Depletion of lamin-A/C leads to mislocalization of pRb and pRb protein family p107 and reduced cell cycle arrest in fibroblasts and the human osteosarcoma cell line U2OS (Johnson et al., 2004). Expression of lamin-A/C also regulates the proteasomal degradation of pRb (Johnson et al., 2004; Dorner et al., 2006), suggesting lamin-A/C has a critical role in stability of pRb and modulation of pRb-associated cell cycle. More recently, *LMNA* existence or mutations affect interaction with binding proteins such as emerin or nesprin-1 (Vaughan et al., 2001; Dilsaver et al., 2018; Sur-Erdem et al., 2020). Homozygous mutation of *LMNA* at Y259X in fibroblasts reveals an abnormal shape of nuclei and mislocalization of the nuclear protein emerin to the endoplasmic reticulum (Muchir et al., 2003). Overexpression of wild-type lamin-A/C in *LMNA*-mutated (nonsense Y259X mutation) fibroblasts restores the localization of nuclear membrane proteins emerin and nesprin-1α (Muchir et al., 2003). Emerin and lamins are implicated in cardiac and muscle-related diseases, as well as cancer metastasis (Mislow et al., 2002; Holaska, 2008; Liddane and Holaska, 2021). Additionally, lamin mutations (*Lmna* knock-out, *Lmna* ^N195K^, *Lmna* ^H222P^, *LMNA* ^R249W^, *LMNA* ^G449V^, *LMNA* ^L489P^, *LMNA* ^W514R^, *LMNA* ^R453W^, and *LMNA* ^R541P^) induce rupture of nuclear envelope and DNA damage in skeletal muscles of mouse models and human biopsy samples with *LMNA* muscular dystrophy (Earle et al., 2020). Although the roles of lamin protein in nuclear morphology and cell cycle regulation are summarized in this section, its categorization is convergent, hence, we separately and more precisely discussed the aging-related role of lamin in next section cellular senescence.

### 2.2 Cellular senescence

Patients with *LMNA* mutation, caused by most frequently nucleotide substitution (1824C to T), show accelerated aging, potentially caused by gradual changes in nuclear structure and aberrant lamin-mediated functions such as nuclear organization and assembly (Goldman et al., 2004). *Lmna* homozygous mutant mice (*Lmna* ^L530P/L530P^) show a broad range of laminopathy symptoms, such as premature aging appearances including hyperkeratosis, bone loss, and decreased hair follicle density (Mounkes et al., 2003), which are similar to those of patients with progeria. Human fibroblasts with *LMNA* ^D47Y^, *LMNA* ^L92F^, *LMNA* ^L387V^, *LMNA* ^R399H^, *LMNA* ^L421P^, and *LMNA* ^R482W^, which accumulate prelamin-A, also enhance oxidative stress and mediate premature features (Caron et al., 2007). Large scale genome mapping in laminar interaction reveals several linker proteins such as LAP2α, which is associated with telomeres (Dechat et al., 2004; De Vos et al., 2010). Damage to telomeres, the physical ends of chromosomal DNA, activates production of mutant *LMNA* gene progerin, and induces a broad range of changes in various genes such as cytoskeleton or cell cycle-related genes in JH-1 and JH-2 fibroblast cells (Cao et al., 2011). In addition, during senescence of human MSCs with high passage, nuclear laminar proteins, including lamin-A, exacerbate the production of telomere aggregates (Raz et al., 2008), suggesting that the lamin and telomere complex are involved in nuclear structural organization. Lamins are also involved in the modulation of telomere homeostasis. During the investigation of interaction between telomere dynamics and *LMNA* mutation, *LMNA* ^R133L^ or *LMNA* ^L140R^ mutations lead to genomic instability and degradation of telomeric repeat-binding factor 2 (TRF2) and accelerate telomere shortening (Saha et al., 2013). It is also identified that its physical interaction between TRF2 and lamin-A/C is required interstitial telomeric loops at chromosome ends (Smith et al., 2018). Lamin-A/C also affects telomere position and long linker region (aa286-488, referred as udTRF2) of TRF2, an essential regulatory domain to interact with lamins (Travina et al., 2021). Telomere shorting or DNA damage is considered the downstream of oxidative stress and subsequent process of cellular senescence (Hutchison, 2011; Sieprath et al., 2012). Thus, cellular and tissue longevity is closely related to functional or structural stability of lamins. Additionally, structural features such as bone loss or enhanced fat mass are associated with aging-related features, we separated the effect of lamin on the differentiation of bone and adipocytes and extensively discuss in Section 3. We summarized the lamin-A/C mutations and its related mechanism (Table 1).

**TABLE 1:** Summary of lamin-A/C mutations and related mechanisms.

Mutation type	Related mechanism	References
Depleted *Lmna*	Mislocalization of pRb → Reduction of cell cycle arrest	Johnson et al. (2004); Dorner et al. (2006)
Depleted *Lmna*, *Lmna* ^N195K^, *Lmna* ^H222P^, *LMNA* ^R249W^, *LMNA* ^G449V^, *LMNA* ^L489P^, *LMNA* ^W514R^, *LMNA* ^R453W^, *LMNA* ^R541P^	Rupture of nuclear envelope and DNA damage in skeletal muscles	Earle et al. (2020)
*LMNA* ^R133L^ *LMNA* ^L140R^	Degrade TRF2 and acceleration of telomere shortening	Saha et al. (2013)
*LMNA* ^D47Y^, *LMNA* ^L92F^, *LMNA* ^L387V^, *LMNA* ^R399H^, *LMNA* ^L421P^, *LMNA* ^R482W^	Accumulate prelamin-A → Enhanced oxidative stress and premature features	Caron et al. (2007)

^a^ Abbreviations: pRB, retinoblastoma protein; TRF2, Telomeric repeat-binding factor 2.

Several studies have addressed that tumor cells possess a mechanism that is protected by cellular senescence, which is considered an onco-suppression process. For example, the involvement of the nuclear envelope protein nestin, which stabilizes lamin-A/C through interaction with the rod domain of nestin, is reported against proteasomal degradation of lamin-A/C in non-small-cell lung carcinoma A549 cells (Zhang et al., 2018). Knockdown of nestin enhances cellular senescence, whereas increased lamin-A/C levels in nestin-null cells reduce cellular senescence (Zhang et al., 2018). The mutant allele at L647R of prelaminar-A as well as the S22A mutant of progerin accelerates tumor cell senescence (Moiseeva et al., 2015). Inoculation of the S22A mutant of progerin into nude mice inhibits tumor progression through impaired mitosis and induces cell senescence (Moiseeva et al., 2015). Cellular senescence is considered as an inhibitory effect on tumor progression; however, it also has both pro- and anti-tumor effects (Campisi, 2013). Although the approach to nuclear lamina protein might be an attractive strategy to investigate the aging process, an anti-tumor strategy through the regulation of cellular senescence should be carefully considered and evaluated in future studies.

### 2.3 Modulatory roles of lamin on cellular signaling and inflammation

The roles of lamin in gene expression and regulation of transcriptional activity have been extensively reviewed, and diverse roles of lamin have been reported in signal transduction (Zastrow et al., 2004; Broers et al., 2006; Heessen and Fornerod, 2007; Marmiroli et al., 2009). Briefly, lamin-A acts as a binding partner with several signaling molecules, such as c-Fos, and its interaction negatively regulates the activity of the activating protein-1 transcription factor (Ivorra' et al., 2006). Lamin-A also binds to extracellular signal-regulated kinase (ERK)1/2 (Gonzalez et al., 2008) and protein phosphatase PP2Aa (Van Berlo et al., 2005).

In addition, laminopathies on inflammatory signaling are addressed as shown in aging-dependent pathologies (Afonso et al., 2016; Tran et al., 2016). *LMNA* mutations such as *LMNA* ^D47Y^, *LMNA* ^R133L^, and *LMNA* ^R482W^ promote the mRNA expression of inflammatory cytokines such as interleukin (IL)-6, IL-33, and monocyte chemoattractant protein (MCP)-1 in vascular smooth muscle cells (Afonso et al., 2016). *Lmna* ^G608G/G608G^ mice also reveal the enhanced serum IL-6, chemokine C-X-C motif ligand-1, and tumor necrosis factor-α (Osorio et al., 2012). Mice with overexpressed *Lmna* mutation (G608G) show several inflammatory cytokine genes such as S100A8 (known as myeloid-related protein MRP8), S100A9 (known as MRP14), small proline-rich protein 2D, and IL-1 receptor antagonist in skin (McKenna et al., 2014). Although accumulating evidences reflect the relationship between laminopathies and inflammation, the precise mechanism is still not known. Thus, roles of lamin on inflammatory signaling should be elucidated beyond one of aging-associated phenotypes.

## 3 Roles of lamin-A/C on aging features

### 3.1 Adipogenesis in MSC and muscle

In an effort to discover the binding partners of lamins, a yeast two-hybrid screening technique reveals the involvement of sterol response element binding protein 1 (SREBP1) and SREBP2 in mouse adipocyte 3T3-L1 cells (Lloyd et al., 2002). Among SREBP isoforms, SREBP1 is a specific isoform of adipocytes, and lamin-A binds to SREBP1, especially to its N-terminal transcription factor domain (Lloyd et al., 2002). This interaction can be explained by that laminopathy with fat deficiency is caused by a reduced interaction between SREBP1 and lamin-A (Lloyd et al., 2002). Polypeptides of SREBP1 reveal the signal sequence for modulation of localization in the nucleus. Wild type lamin A and its gene variants bind to the SREBP1 signal sequence and cause SREBP1 localization (Duband-Goulet et al., 2011). In addition, as a genetic disorder of Dunnigan-type familial partial lipodystrophy, *LMNA* ^R482W^ fails to regulate SREBP1 activity (Vadrot et al., 2015).

**FIGURE 4:**
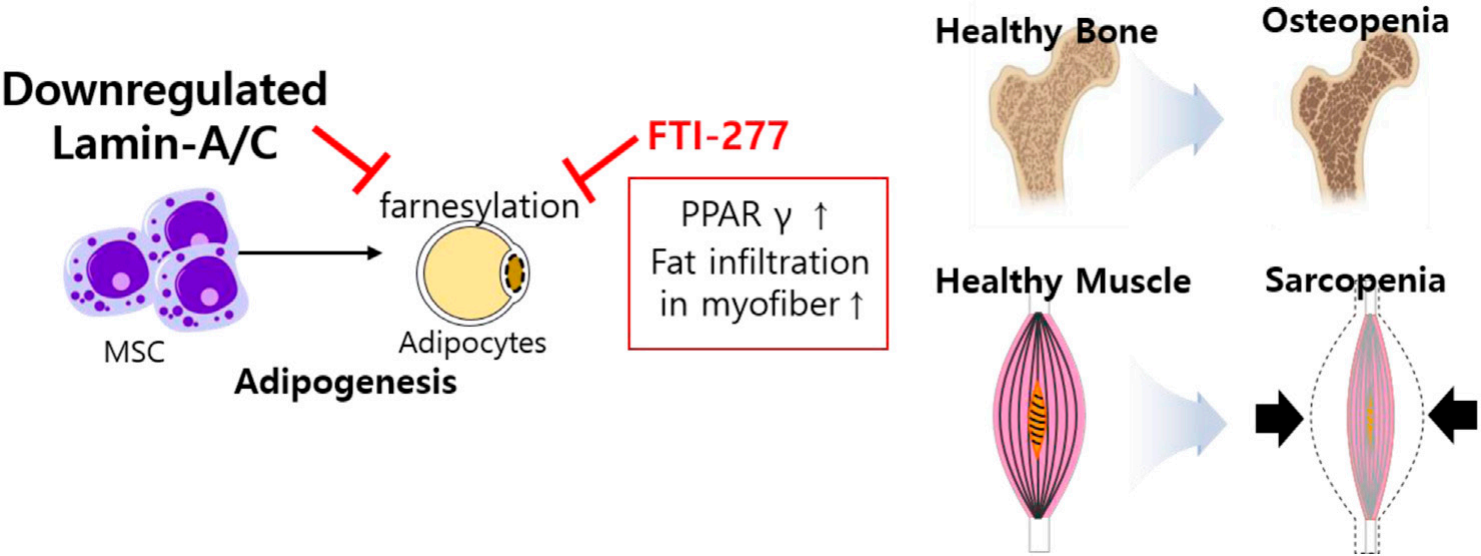
Schematic illustration of aberrantly expressed lamin-A/C in MSCs. Downregulated lamin-A/C induces adipogenesis and aging feature such as osteopenia and sarcopenia. MSC, mesenchymal stem cell; PPARγ, peroxisome proliferator activator gamma.

Differentiated cells are required protein farnesylation for adipogenesis, which is differentiation process of white fat into mature adipocytes (Klemm et al., 2001). The inhibition of farnesylation with farnesyltransferase inhibitor (FTI)-277 arrests MSC differentiation, enhances prelamin-A and subsequently reduces SREBP1 expression, peroxisome proliferator activator gamma (PPARγ) expression, and PPARγ activity during bone marrow adipogenesis (Rivas et al., 2007). The role of lamins has also been addressed in osteopenia and sarcopenia through the regulation of adipocyte differentiation (Tong et al., 2011). *Lmna*-null mice and *Lmna* ^H222P/H222P^ mice show reduced WNT/β-catenin signaling, suggesting that lamins are involved in muscle and bone strength (Le Dour et al., 2017) and defects in lamins enhance adipogenic factors such as PPARγ and fat infiltration in myofibers (Tong et al., 2011). These studies address that the loss of lamin mediates frailty syndrome-related sarcopenia and osteopenia and reflects consistent features of aging (Figure 4).

### 3.2 Bone remodeling

**FIGURE 5:**
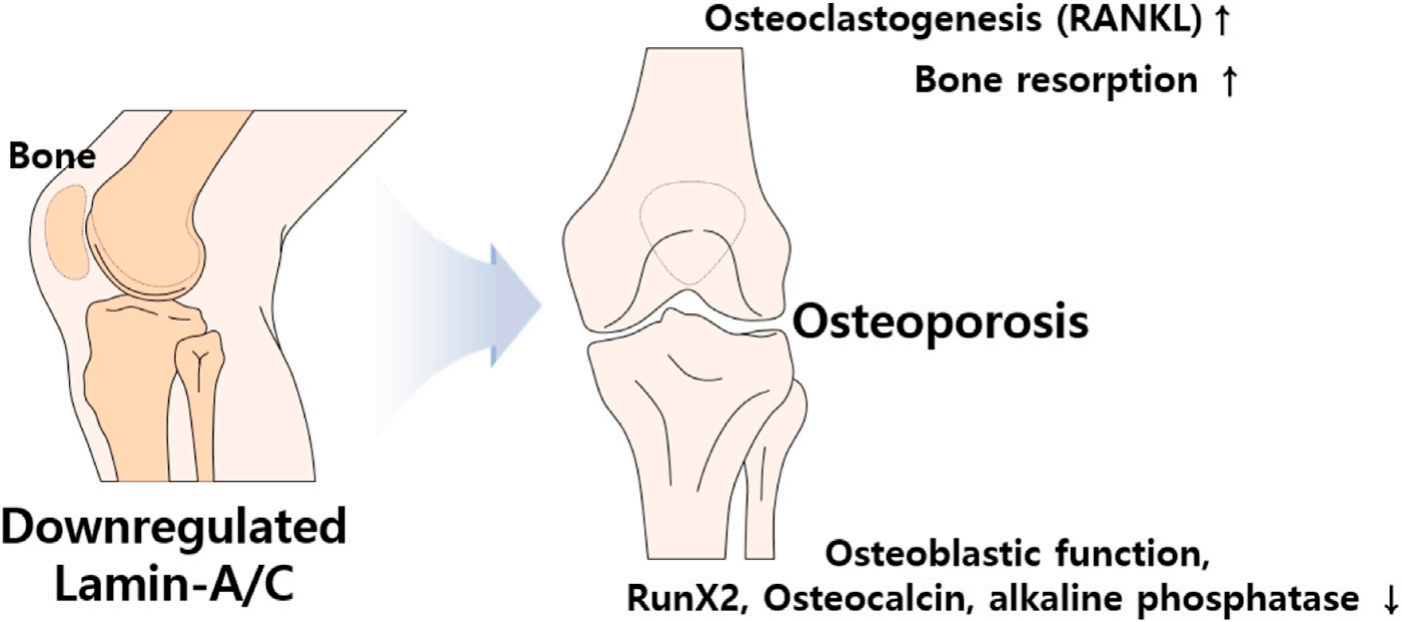
Schematic illustration of aberrantly expressed lamin-A/C in bone system. Abnormal expression of lamin induces osteoporosis which caused by upregulated RANKL and bone resorption and downregulated osteoblastic function, RunX2, osteocalcin, and phosphatase activity. RANKL, Receptor activator of NF-κB ligand; Runx2, Runt-related transcription factor 2.

The HGP syndrome is characterized by premature osteoporosis and reduced bone mass. It was also assumed that the lamin-A/C mutation is involved in osteoblast differentiation. Knockdown of lamin-A/C reduces osteoblastogenetic process of MSCs and reduces osteoblast differentiation markers Runt-related transcription factor 2 (Runx2) and osteocalcin levels as well as alkaline phosphatase activity, whereas it enhances osteoclastogenesis marker protein receptor activator of NF-κB ligand (RANKL)/osteoprotegerin ratio and tartrate-resistant acidic phosphatase (TRAP)-positive staining (Akter et al., 2009; Rauner et al., 2009). Bone remodeling is a coordinated process between osteoblasts and osteoclasts. Impaired osteoblastic function and osteoclastogenesis lead to changes in bone content. Thus, bone formation by osteoblasts and bone resorption by active osteoclasts are essential homeostatic processes in bone remodeling (Kang et al., 2020). Differentiated osteoclasts, also known as osteoclastogenesis, possess bone resorption activity through the stimulation of RANKL and macrophage colony-stimulating factor (Yasuda et al., 1998). Accordingly, reduced lamin-A/C facilitates the process of osteoporosis such as inhibited osteoblastic function, enhanced osteoclastogenesis, and increased bone resorption area (Rauner et al., 2009). Lamin-A/C–deficient animal studies have also addressed the inhibition of osteoblastic differentiation and the reduction in bone formation (Li et al., 2011). Thus, lamin-A/C is required for osteoblast differentiation and normal bone biogenesis (Li et al., 2011; Vidal et al., 2012). *LMNA-*overexpressing human MSCs enhance osteoblastic differentiation of MSCs, the level of nuclear β-catenin, and its transcriptional activity, whereas knockdown of lamin-A/C with siRNA dramatically reduces nuclear β-catenin levels (Bermeo et al., 2015). In addition, adipocyte differentiation is attenuated by *LMNA* overexpression in human MSCs (Bermeo et al., 2015). Above all, loss of lamin-A/C mediates reduced osteoblasts differentiation and enhances adipogenic differentiation of MSCs (Figure 5).

### 3.3 Keratinocyte differentiation

The essential role of lamin-A/C in keratinocyte differentiation has been described in several studies. For example, the HGP syndrome mutation reveals skin phenotypes such as hyperplastic epidermis feature, inflammatory response in dermal layer, and hypodermal fat layer (McKenna et al., 2014) and mice with *Lmna* ^G608G^ mutation reveals abnormal skin development (Sagelius et al., 2008). Knockdown of Akt1 in mouse keratinocytes enhances lamin-A/C expression (Naeem et al., 2015). Degradation of lamin-A/C is prevented by blocking Akt function. Nuclear lamin is retained by preventing phosphorylation at Akt phosphorylation sites, S404 and S301 (Bertacchini et al., 2013). The S301 mutant reveals a reduction in keratinocyte differentiation markers such as loricrin, keratin 1/10, and filaggrin, and enhances the expression of bone morphogenetic protein 2 (BMP2)/Smad1 (Naeem et al., 2015). The terminal differentiation of keratinocytes requires nuclear degradation in the cornified layer of the skin. Thus, the retention of nuclear lamin is involved in parakeratosis, such as enhanced BMP2 signaling, which is observed in psoriasis, eczema, and atopic dermatitis (Sakurai et al., 2002). More recently, it has been addressed that keratinocyte differentiation is required Akt1-dependent nuclear lamin A/C distribution (Rogerson et al., 2021).

**FIGURE 6:**
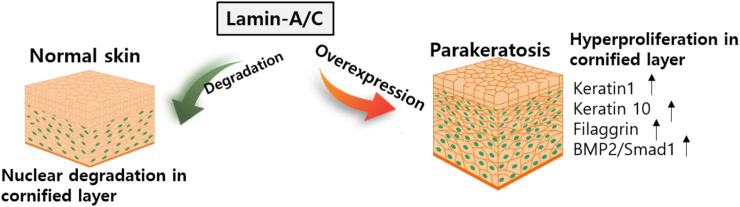
Schematic illustration of lamin-A/C in skin development. Abnormal overexpression of lamin-A/C induces hyperproliferative keratinocyte with upregulated signaling modulators. BMP2, bone morphogenetic protein 2.

Nuclear remodeling is essential process during the keratinocyte differentiation. However, verification of network between nuclear proteins such as lamin-A/C and keratinocyte differentiation has not been extensively studied. Thus, further work is required to determine the precise mechanism of molecular network in various skin diseases. Above all, considering the significance of the differentiation mechanism, the regulatory role of lamin-A/C might be critically associated with chromosomal functions such as regulation of DNA transcription including the maintenance of nuclear envelope. The expression of lamin-A/C is clearly differentially regulated in different tissues and precise verification of lamin modulation in differentiated tissues would be next challengeable issues. We illustrated the role of lamin in keratinocyte differentiation systems (Figure 6).

### 3.4 Cardiac maintenance

**FIGURE 7:**
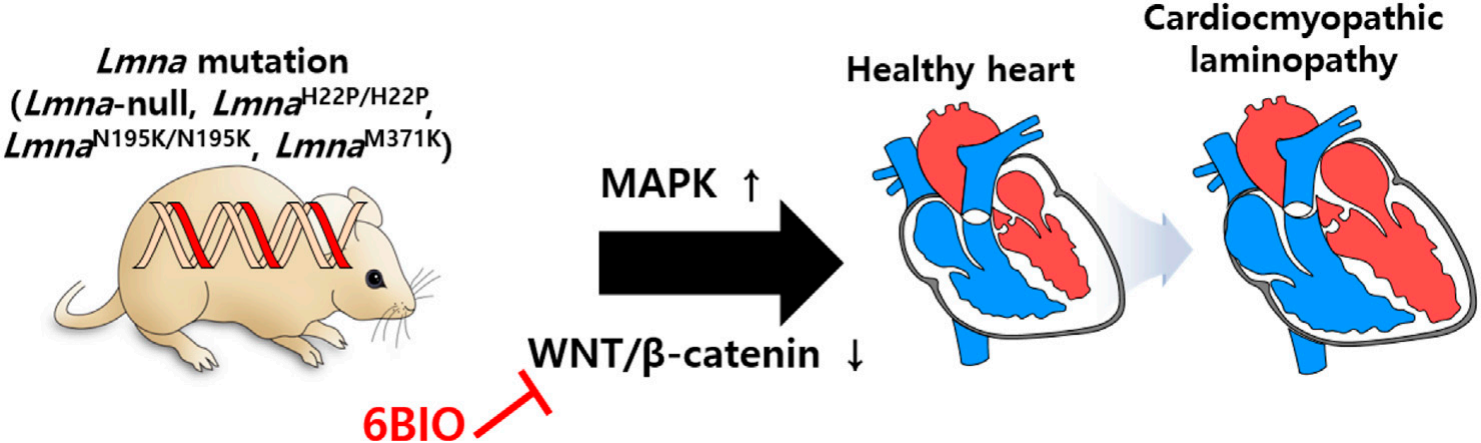
Schematic illustration of lamin mutations in cardiac system. Cardiomyopathy symptoms are occurred by misregulation of MAPK and WNT/β-catenin in mutant mouse models of *Lmna* (*Lmna*-null, *Lmna* ^H222P/H222P^, *Lmna* ^N195K/N195K^, and *Lmna* ^M371K^).

The involvement of lamin-A/C in the cardiac system has also been investigated in various studies. Gene defects of *LMNA* reveal abnormal cardiac function and dilated cardiomyopathy (Bonne et al., 1999; Fatkin et al., 1999; Bonne et al., 2000; Ben Yaou et al., 2006; Jacob and Garg, 2006; Sylvius and Tesson, 2006). To understand laminopathies in cardiac tissue, a mutant mouse model of *Lmna* is developed. Summary of lamin mutations (lamin-null, -mis-sense, -transgenic lines) is addressed (Lu et al., 2011) and is beneficial for understanding various laminopathies. Cardiomyopathy symptoms occur in *Lmna*-null (Sullivan et al., 1999), *Lmna* ^H222P/H222P^ (Arimura et al., 2005; Muchir et al., 2007; Muchir et al., 2012; Le Dour et al., 2017), *Lmna* ^N195K/N195K^ (Mounkes et al., 2005), and *Lmna* ^M371K^ (Wang et al., 2006) lines. With an effort to discover effects of lamin-A mutation on gene expression with genome-wide expression profiles, the involvement of MAPK and its downstream signaling pathway are reported in the cardiomyopathic laminopathy of *Lmna* ^H222P/H222P^ (Muchir et al., 2007). Moreover, WNT/β-catenin signaling is decreased in *Lmna* ^H222P/H222P^ mice, whereas activation of β-catenin signaling by treatment with 6-bromoindirubin-3′-oxime (6BIO, inhibitor of glycogen synthase kinase 3β) to prevent the destruction of β-catenin enhances cardiac function in *Lmna* mutant mice (Le Dour et al., 2017). Restoration of β-catenin signaling by 6BIO treatment protects myocardial dysfunction from aging (Guo et al., 2020). Regulation of WNT/β-catenin signaling is a promising strategy against an aging heart with cardiomyopathy (Figure 7). Aging features owing to defects of lamin function could be modulated by the application of potential signaling regulators. In addition, it has been recently addressed that cytoplasmic intermediate filament protein desmin mis-localizes in cardiomyocytes of *Lmna* ^H222P/H222P^ mice (Galata et al., 2018). Overexpression of heat shock protein αB-crystallin rescues cardiomyopathy with *Lmna* mutation (*Lmna* ^H222P/H222P^) including desminopathy (Galata et al., 2018). These results indicate that cytoplasmic filament protein desmin is associated with nuclear lamin network in cardiomyopathy and αB-crystallin is suggested to rebuild the cytoskeleton-nucleoskeleton network (Galata et al., 2018; Maggi et al., 2021). Although an overview of the management of cardiac disease in *LMNA*-associated laminopathy has been provided (Chen et al., 2019; Kirkland et al., 2023; Wang and Dobreva, 2023), treatment of cardiomyopathy caused by laminopathies is remains a challenging issue.

## 4 Role of lamin-A/C on oncology

Altered nuclear lamin as an oncogenic signaling pathway has been observed in various cancer tissues. Lamin expression in cancerous tissues is highly diverse. It has been reported that loss of lamin-A/C expression in lung (Machiels et al., 1995) and colon cancer cells, which are different from lamin-A/C expressed hepatic tumor cells (Aebi et al., 1986; Hytiroglou et al., 1993). Knockdown of lamin-A/C using shRNA reveals nuclear morphological deformation of primary breast epithelial cells, be similar to phenotype of cancer cells (Capo-chichi et al., 2011b). In cancer system, it is difficult to define high or low levels of lamin-A/C expression. 47% of ovarian cancer lacks lamin-A/C expression (Capo-chichi et al., 2011a). Non-cancerous ovarian epithelial cells show morphological deformities of nuclear shape with deletion of lamin-A/C using small interfering RNA (Capo-chichi et al., 2011a). In addition to ovarian cancer, 17.8% of colon cancer tissue reveals low lamin-A/C expression, whereas 82.2% of colon cancer shows high lamin-A/C expression (Belt et al., 2011). Gastrointestinal cancers (Wu et al., 2009) such as gastric carcinoma show loss of lamin expression, whereas prostate (Kong et al., 2012; Khan et al., 2018) and breast cancers (Zhang and Lv, 2017) show both characteristics, and colorectal cancer (Willis et al., 2008) reveals different patterns of lamin expression. Thus, in this section, although experimental evidences possess limitation, we highlighted individually the tissue-selective expression pattern of lamin-A/C as a negative or positive regulator of cancers.

Immunohistochemical analysis of lamin reveals that the primary isolated colonic and gastric adenocarcinoma tissues show a reduction or absent in nuclear lamin-A/C and -B1 expression and cytoplasmic detection of lamin (Moss et al., 1999). Other tissues, including cervical squamous carcinoma, uterine adenocarcinoma, prostate adenocarcinoma, breast adenocarcinoma, and non-small cell lung carcinoma, also reveal immunohistochemically reduced or absent lamin expression (Moss et al., 1999). Reduced lamin protein levels are considered a common feature in tumor tissues. Additional experimental evidence is available for gastric carcinomas. mRNA and protein expression in addition to immunohistochemical analysis have revealed reduced expression of lamin-A/C in primary gastric carcinoma (Wu et al., 2009). Additionally, loss of lamin enhances migration in ovarian cancer HO-8910 cells (Wang et al., 2019). As a potential negative biomarker, lamin-A/C can be used for the diagnosis of gastric cancer, colon cancer, or ovarian cancer HO-8910 cell lines.

However, as a risk biomarker, several experimental evidences on lamin expression have revealed the opposite. More recently, although sample size is restricted, deep hybrid machine learning technique is revealed that nuclear A and B type of lamins are newly addressed as potent diagnostic markers of ovarian cancers (Sengupta et al., 2022). Moreover, lamin-A/C is expressed in colonic stem cells and the ectopically expressed lamin-A colorectal cancer cell line SW480 shows enhanced morphological changes and invasive or motile properties (Willis et al., 2008). GFP-tagged lamin-A enhances actin-associated protein T-plastin and reduces cellular adhesive protein E-cadherin in SW480 cells (Willis et al., 2008). As shown in colorectal cancer, Kong *et al.* reported that enhanced lamin-A/C expression increases the risk of invasive characteristics of prostate cancer through the involvement of the phosphoinositide 3-kinase (PI3K)/Akt signaling pathway (Kong et al., 2012). These results indicate that lamin-A/C–positive colorectal and prostate cancers possess stem cell-like features.

It has been demonstrated that lack of lamin-A/C was revealed in human leukemia cell HL-60 (Paulin-Levasseur et al., 1988), early-stage lymphocytes (Guilly et al., 1990), lymphoblastic leukemia, and non-Hodgkin’s lymphoma cells (Stadelmann et al., 1990). However, more recently, lamin-A/C is expressed in CD30 (+) Reed-Sternberg cells from Hodgkin’s lymphoma, not in CD20 (+) non-neoplastic lymphocytes B (Jansen et al., 1997). Additionally, three-dimensional structure of lamin was identified in Reed-Sternberg cells (Contu et al., 2018).

Several evidences are limitedly provided or experimental cellular states are clearly undefined in oncology. Why does the expression of lamin play different roles in different cancers and is this expression of lamin dependent on the motility of cancer cells? Based on current experimental evidences, type of cancers and cancerous circumstances may reflect lamin expression. Especially, the cell suspension state has been shown to modulate lamin-A/C expression. Suspended breast cancer cells promote reattachment through enhanced lamin-A/C, suggesting that the cellular status may provide a broad spectrum of lamin and cytoskeletal proteins (Zhang and Lv, 2017; Ovsiannikova et al., 2021; Zhao et al., 2022).

Therefore, type of cancer or physical cellular state should be carefully considered on the aspect of laminopathies and the diversity of the role of lamins in various cancers also should be considered in future studies. Above all, although various roles of nuclear lamin-A/C in human diseases are highlighted in this review, potential roles of lamin in human diseases is not limited to several specific issues.

## 5 Various modulating factors and effector signals on lamin-A/C expression

Understanding the relationship between lamin and signaling modulators might be an effective strategy for various laminopathies-associated pathogenesis, which are currently no therapy strategy. Thus, modulatory mechanism or effector signals of lamin-associated biology are elucidated in this section. Using proteomic-based analysis, the substrates of the serine/threonine kinase Akt are identified as the modulating factor of lamin expression (Cenni et al., 2008). Akt phosphorylates the lamin-A precursor prelamin-A at Ser404 and modulates the transcription of *Lmna* (Cenni et al., 2008; Bertacchini et al., 2013). Phosphorylation of lamin-A/C at Ser404 is mediated by insulin treatment, whereas no phosphorylation responses to Akt stimulation are triggered in primary fibroblast from patient with *LMNA* ^R401C^-EDMD2 (Cenni et al., 2008). Its Akt-mediated lamin phosphorylation is critical for lamina degradation. Akt/protein kinase B (PKB) signaling regulates both *Lmna* and prelamin-A stability (Bertacchini et al., 2013; Rogerson et al., 2021). In contrast, the Akt1-depleted cornified layer restores lamin-A/C expression and mediates parakeratosis, which is abnormal retention of nuclei, in cornified layer (Naeem et al., 2015).

Farnesylation process also regulates lamin-A/C expression. Accumulated farnesylation of prelamin-A is associated with genetic diseases in mice with HGP syndrome mutation *Lmna* ^G608G^ (Yang et al., 2005; Yang et al., 2008). Application of farnesyltransferase inhibitor FTI-277 reduces prelamin-A expression and adipogenic differentiation in bone marrow MSCs (Rivas et al., 2007) and ABT-100 also reduces rib bone fracture and enhances the survival of mice with *Lmna* ^G608G^ (Yang et al., 2008).

In addition to farnesylation, matrix microenvironment influences cellular motility and differentiation. Matrix stiffness and strength modulate lamin expression. The soft matrix phosphorylates lamin-A at Ser22, Ser390, Ser404, and Thr424 (Swift et al., 2013). Matrix stiffness is associated with myosin II activity, and lamin-A/C expression is enhanced by reduced nuclear stress (Buxboim et al., 2014). Reduced stiffness dephosphorylates lamin-A/C at Ser22, which mediates lamina signaling (Buxboim et al., 2014; Virtanen et al., 2023) and differential expression of lamin-A/C as an extracellular matrix-dependent mechanosensor protein has also been suggested (Ovsiannikova et al., 2021; Urciuoli et al., 2021). More recently, phosphorylation of lamin-A/C at Ser22 modulates voltage-gated Na_V_1.5 channel activity (Olaopa et al., 2021). These results may provide the potent role of lamin on the transitional pattern of adhesion to suspension.

Kinase-dependent lamin-A/C expression is also addressed in several studies. The cyclin-dependent kinase (Cdk) inhibitor RO3306 inhibits the phosphorylation of lamin-A/C and its degradation (Buxboim et al., 2014). The proliferation of vascular smooth muscle cells is affected by cyclic stretching, and its mechanosensitive application modulates nuclear proteins such as emerin and lamin-A/C expression (Qi et al., 2016). Hypertensive mechanical application enhances emerin and lamin-A/C expression, indicating that emerin and lamin-A/C inhibit the hyper-proliferation of vascular smooth muscle cells (Qi et al., 2016). Moreover, nestin depletion leads to the activation of Cdk5, which is involved in the phosphorylation and degradation of lamin-A/C (Zhang et al., 2018). The oncogenic focal adhesion kinase (FAK) is also involved in cellular senescence-related signaling. Inhibition of FAK with its enzymatic inhibitor PF-573228 enhances p53 expression and reduces lamin-A/C expression in lung cancer cells (Chuang et al., 2019). FAK-downregulated p53 expression is related to cellular senescence, and FAK inhibition enhances β-galactosidase activity, which is a cellular senescence marker (Chuang et al., 2019). Thus, modulation of FAK signaling mediates changes in lamin-A/C expression and cellular senescence programs in lung cancer.

As another process of lamin modulation, the lamin structure is cleaved by caspase-6, and the subsequent lamin cleavage induces apoptosis in colon cancer cells (Lee et al., 2006). Recently, cyclic stretch–induced microRNA-214-3p is shown to bind to *Lmna* and reduces lamin-A/C protein expression, and subsequently induces the apoptosis of vascular smooth muscle cells (Bao et al., 2020). Treatment with dexamethasone is shown to enhance lamin-A/C nuclear content in ataxia telangiectasia fibroblasts (Ricci et al., 2021). More recently, we addressed that histamine-mediated calcium/calmodulin-dependent kinase II activation and reactive oxygen species generation are involved in the enhancement of lamin-A/C expression in lung adenocarcinoma cell line A549 (Kim et al., 2022). We presented a summary of effector signals and molecules on lamin-A/C expression in Table 2 and future detailed investigations could provide the regulatory mechanism of nuclear lamin biology to improve the current knowledge of lamin pathophysiology on cellular and tissue longevity.

**TABLE 2:** Summary of effector signals and molecules on lamin expression.

Related mechanism		Effect on lamin modulation	References
Intracellular Ca^2+^ increase/CaMKII		Enhanced lamin-A/C in adenocarcinoma cell line	Kim et al. (2022)
Oxidative stress (dopaminergic neurotoxin 6-hydroxydopamine)		Induced lamin phosphorylation	Nakamura et al. (2006)
Akt/PKB		Induced lamin phosphorylation	Bertacchini et al. (2013); Naeem et al. (2015)
Inhibition of farnesylation (FTI-277, ABT-100)		Inhibited lamin-A/C phosphorylation	Rivas et al. (2007); Yang et al. (2008)
Inhibition of Cdk (RO3306)		Inhibited lamin-A/C phosphorylation	Buxboim et al. (2014)
Inhibition of FAK (PF-573228)		Reduced lamin-A/C expression	Chuang et al. (2019)
Matrix stiffness		Soft matrix enhanced lamin-A/C expression	Buxboim et al. (2014); Urciuoli et al. (2021)
Cell suspension state		Enhanced lamin-A/C in breast cancer	Zhang and Lv (2017); Zhao et al. (2022)
Cyclic stretch-induced miR-214-3p		Reduced lamin-A/C protein expression	Bao et al. (2020)
Hypertensive mechanical application		Enhancement of lamin-A/C expression in VSMC	Qi et al. (2016)

^a^ Abbreviations: Akt/PKB, Akt/protein kinase B; cdk, cyclin-dependent kinase; FAK, focal adhesion kinase; VSMC, vascular smooth muscle cell; miR, microRNA; Ca/CaMKII, Calcium/Calmodulin-dependent protein kinase II.

## 6 In conclusion and perspectives

Lamin proteins regulate various nuclear activities that are implicated in the nuclear architecture, cellular senescence, differentiation, and signaling modulation. Lamins also possess attractive roles in numerous cellular mechanisms, such as aging, tumor suppression, maintenance of nuclear shape, and sensing of extracellular matrix stiffness. Although lamin-associated disease features share several oxidative process such as cellular senescence, posttranslational modification of lamin is also affected by oxidative stress (Eaton et al., 2003; Pekovic et al., 2011). Although various experimental evidences are addressed, precise evidences and molecular mechanism still remain verification. Further studies of laminar signaling continue to present several questions and challenges with regard to crosstalk between aging phenotype, differentiation, and cellular redox homeostasis. Moreover, extracellular matrix, cellular stress, and kinase-dependent mechanism are illuminated and gradually developed in lamin modulation. Thus, regulatory signals and treatment drugs for lamin modulation, compositional changes in lamin, and precise and differential roles of lamin in various cancer cells are ongoing issues. Thus, understanding nuclear lamin biology as an attractive decoding tool is the Rosetta stone of cellular longevity-associated human diseases, including aging, redox potential, or modulation of tumorigenesis might provide extended therapeutic applications on human longevity.

## References

[B1] Aaronson R. P. Blobel G. (1975). Isolation of nuclear pore complexes in association with a lamina. Proc. Natl. Acad. Sci. U. S. A. 72, 1007–1011. 10.1073/pnas.72.3.1007 1055359 PMC432453

[B2] Aebi U. Cohn J. Buhle L. Gerace L. (1986). The nuclear lamina is a meshwork of intermediate-type filaments. Nature 323, 560–564. 10.1038/323560a0 3762708

[B3] Afonso P. Auclair M. Boccara F. Vantyghem M. C. Katlama C. Capeau J. (2016). LMNA mutations resulting in lipodystrophy and HIV protease inhibitors trigger vascular smooth muscle cell senescence and calcification: Role of ZMPSTE24 downregulation. Atherosclerosis 245, 200–211. 10.1016/j.atherosclerosis.2015.12.012 26724531

[B4] Ahn J. Jo I. Kang S. M. Hong S. Kim S. Jeong S. (2019). Structural basis for lamin assembly at the molecular level. Nat. Commun. 10, 3757. 10.1038/s41467-019-11684-x 31434876 PMC6704074

[B5] Akter R. Rivas D. Geneau G. Drissi H. Duque G. (2009). Effect of lamin A/C knockdown on osteoblast differentiation and function. J. Bone Min. Res. 24, 283–293. 10.1359/jbmr.081010 18847334

[B6] Arimura T. Helbling-Leclerc A. Massart C. Varnous S. Niel F. Lacene E. (2005). Mouse model carrying H222P-Lmna mutation develops muscular dystrophy and dilated cardiomyopathy similar to human striated muscle laminopathies. Hum. Mol. Genet. 14, 155–169. 10.1093/hmg/ddi017 15548545

[B7] Bao H. Li H. P. Shi Q. Huang K. Chen X. H. Chen Y. X. (2020). Lamin A/C negatively regulated by miR-124-3p modulates apoptosis of vascular smooth muscle cells during cyclic stretch application in rats. Acta Physiol. (Oxf) 228, e13374. 10.1111/apha.13374 31495066

[B8] Baricheva E. A. Berrios M. Bogachev S. S. Borisevich I. V. Lapik E. R. Sharakhov I. V. (1996). DNA from *Drosophila melanogaster* beta-heterochromatin binds specifically to nuclear lamins *in vitro* and the nuclear envelope *in situ* . Gene 171, 171–176. 10.1016/0378-1119(96)00002-9 8666268

[B9] Belt E. J. Fijneman R. J. Van Den Berg E. G. Bril H. Delis-Van Diemen P. M. Tijssen M. (2011). Loss of lamin A/C expression in stage II and III colon cancer is associated with disease recurrence. Eur. J. Cancer 47, 1837–1845. 10.1016/j.ejca.2011.04.025 21621406

[B10] Ben Yaou R. Gueneau L. Demay L. Stora S. Chikhaoui K. Richard P. (2006). Heart involvement in lamin A/C related diseases. Arch. Mal. Coeur Vaiss. 99, 848–855. 17067107

[B11] Bermeo S. Vidal C. Zhou H. Duque G. (2015). Lamin A/C acts as an essential factor in mesenchymal stem cell differentiation through the regulation of the dynamics of the wnt/β-catenin pathway. J. Cell. Biochem. 116, 2344–2353. 10.1002/jcb.25185 25846419

[B12] Bertacchini J. Beretti F. Cenni V. Guida M. Gibellini F. Mediani L. (2013). The protein kinase Akt/PKB regulates both prelamin A degradation and Lmna gene expression. FASEB J. 27, 2145–2155. 10.1096/fj.12-218214 23430973

[B13] Bonne G. Di Barletta M. R. Varnous S. Becane H. M. Hammouda E. H. Merlini L. (1999). Mutations in the gene encoding lamin A/C cause autosomal dominant Emery-Dreifuss muscular dystrophy. Nat. Genet. 21, 285–288. 10.1038/6799 10080180

[B14] Bonne G. Mercuri E. Muchir A. Urtizberea A. Becane H. M. Recan D. (2000). Clinical and molecular genetic spectrum of autosomal dominant Emery-Dreifuss muscular dystrophy due to mutations of the lamin A/C gene. Ann. Neurology 48, 170–180. 10.1002/1531-8249(200008)48:2<170::aid-ana6>3.0.co;2-j 10939567

[B15] Broers J. L. V. Ramaekers F. C. S. Bonne G. Ben Yaou R. Hutchison C. J. (2006). Nuclear lamins: Laminopathies and their role in premature ageing. Physiol. Rev. 86, 967–1008. 10.1152/physrev.00047.2005 16816143

[B16] Buxboim A. Swift J. Irianto J. Spinler K. R. Dingal P. C. Athirasala A. (2014). Matrix elasticity regulates lamin-A,C phosphorylation and turnover with feedback to actomyosin. Curr. Biol. 24, 1909–1917. 10.1016/j.cub.2014.07.001 25127216 PMC4373646

[B17] Campisi J. (2013). Aging, cellular senescence, and cancer. Annu. Rev. Physiol. 75, 685–705. 10.1146/annurev-physiol-030212-183653 23140366 PMC4166529

[B18] Cao K. Blair C. D. Faddah D. A. Kieckhaefer J. E. Olive M. Erdos M. R. (2011). Progerin and telomere dysfunction collaborate to trigger cellular senescence in normal human fibroblasts. J. Clin. Invest. 121, 2833–2844. 10.1172/JCI43578 21670498 PMC3223819

[B19] Capo-Chichi C. D. Cai K. Q. Simpkins F. Ganjei-Azar P. Godwin A. K. Xu X. X. (2011a). Nuclear envelope structural defects cause chromosomal numerical instability and aneuploidy in ovarian cancer. BMC Med. 9, 28. 10.1186/1741-7015-9-28 21439080 PMC3072346

[B20] Capo-Chichi C. D. Cai K. Q. Smedberg J. Ganjei-Azar P. Godwin A. K. Xu X. X. (2011b). Loss of A-type lamin expression compromises nuclear envelope integrity in breast cancer. Chin. J. Cancer 30, 415–425. 10.5732/cjc.010.10566 21627864 PMC3941915

[B21] Caron M. Auclair M. Donadille B. Bereziat V. Guerci B. Laville M. (2007). Human lipodystrophies linked to mutations in A-type lamins and to HIV protease inhibitor therapy are both associated with prelamin A accumulation, oxidative stress and premature cellular senescence. Cell Death Differ. 14, 1759–1767. 10.1038/sj.cdd.4402197 17612587

[B22] Cenni V. Bertacchini J. Beretti F. Lattanzi G. Bavelloni A. Riccio M. (2008). Lamin A Ser404 is a nuclear target of Akt phosphorylation in C2C12 cells. J. Proteome Res. 7, 4727–4735. 10.1021/pr800262g 18808171

[B23] Chen S. N. Sbaizero O. Taylor M. R. G. Mestroni L. (2019). Lamin A/C cardiomyopathy: Implications for treatment. Curr. Cardiol. Rep. 21, 160. 10.1007/s11886-019-1224-7 31773301

[B24] Chuang H. H. Wang P. H. Niu S. W. Zhen Y. Y. Huang M. S. Hsiao M. (2019). Inhibition of FAK signaling elicits lamin A/C-associated nuclear deformity and cellular senescence. Front. Oncol. 9, 22. 10.3389/fonc.2019.00022 30761269 PMC6363943

[B25] Contu F. Rangel-Pozzo A. Trokajlo P. Wark L. Klewes L. Johnson N. A. (2018). Distinct 3D structural patterns of lamin A/C expression in Hodgkin and reed-sternberg cells. Cancers (Basel) 10, 286. 10.3390/cancers10090286 30149530 PMC6162537

[B26] Crisp M. Burke B. (2008). The nuclear envelope as an integrator of nuclear and cytoplasmic architecture. Febs Lett. 582, 2023–2032. 10.1016/j.febslet.2008.05.001 18474238

[B27] De Vos W. H. Houben F. Hoebe R. A. Hennekam R. Van Engelen B. Manders E. M. M. (2010). Increased plasticity of the nuclear envelope and hypermobility of telomeres due to the loss of A-type lamins. Biochimica Biophysica Acta-General Subj. 1800, 448–458. 10.1016/j.bbagen.2010.01.002 20079404

[B28] Dechat T. Gajewski A. Korbei B. Gerlich D. Daigle N. Haraguchi T. (2004). LAP2alpha and BAF transiently localize to telomeres and specific regions on chromatin during nuclear assembly. J. Cell Sci. 117, 6117–6128. 10.1242/jcs.01529 15546916

[B29] Dechat T. Pfleghaar K. Sengupta K. Shimi T. Shumaker D. K. Solimando L. (2008). Nuclear lamins: Major factors in the structural organization and function of the nucleus and chromatin. Genes Dev. 22, 832–853. 10.1101/gad.1652708 18381888 PMC2732390

[B30] Dilsaver M. R. Chen P. Thompson T. A. Reusser T. Mukherjee R. N. Oakey J. (2018). Emerin induces nuclear breakage in Xenopus extract and early embryos. Mol. Biol. Cell 29, 3155–3167. 10.1091/mbc.E18-05-0277 30332321 PMC6340207

[B31] Dorner D. Vlcek S. Foeger N. Gajewski A. Makolm C. Gotzmann J. (2006). Lamina-associated polypeptide 2alpha regulates cell cycle progression and differentiation via the retinoblastoma-E2F pathway. J. Cell Biol. 173, 83–93. 10.1083/jcb.200511149 16606692 PMC2063793

[B32] Duband-Goulet I. Woerner S. Gasparini S. Attanda W. Konde E. Tellier-Lebegue C. (2011). Subcellular localization of SREBP1 depends on its interaction with the C-terminal region of wild-type and disease related A-type lamins. Exp. Cell Res. 317, 2800–2813. 10.1016/j.yexcr.2011.09.012 21993218 PMC3215798

[B33] Earle A. J. Kirby T. J. Fedorchak G. R. Isermann P. Patel J. Iruvanti S. (2020). Mutant lamins cause nuclear envelope rupture and DNA damage in skeletal muscle cells. Nat. Mater 19, 464–473. 10.1038/s41563-019-0563-5 31844279 PMC7102937

[B34] Eaton P. Jones M. E. Mcgregor E. Dunn M. J. Leeds N. Byers H. L. (2003). Reversible cysteine-targeted oxidation of proteins during renal oxidative stress. J. Am. Soc. Nephrol. 14, S290–S296. 10.1097/01.asn.0000078024.50060.c6 12874448

[B35] Eriksson M. Brown W. T. Gordon L. B. Glynn M. W. Singer J. Scott L. (2003). Recurrent de novo point mutations in lamin A cause Hutchinson-Gilford progeria syndrome. Nature 423, 293–298. 10.1038/nature01629 12714972 PMC10540076

[B36] Evangelisti C. Rusciano I. Mongiorgi S. Ramazzotti G. Lattanzi G. Manzoli L. (2022). The wide and growing range of lamin B-related diseases: From laminopathies to cancer. Cell Mol. Life Sci. 79, 126. 10.1007/s00018-021-04084-2 35132494 PMC8821503

[B37] Fatkin D. Macrae C. Sasaki T. Wolff M. R. Porcu M. Frenneaux M. (1999). Missense mutations in the rod domain of the lamin A/C gene as causes of dilated cardiomyopathy and conduction-system disease. N. Engl. J. Med. 341, 1715–1724. 10.1056/NEJM199912023412302 10580070

[B38] Fisher D. Z. Chaudhary N. Blobel G. (1986). cDNA sequencing of nuclear lamins A and C reveals primary and secondary structural homology to intermediate filament proteins. Proc. Natl. Acad. Sci. U. S. A. 83, 6450–6454. 10.1073/pnas.83.17.6450 3462705 PMC386521

[B39] Galata Z. Kloukina I. Kostavasili I. Varela A. Davos C. H. Makridakis M. (2018). Amelioration of desmin network defects by alpha B-crystallin overexpression confers cardioprotection in a mouse model of dilated cardiomyopathy caused by LMNA gene mutation. J. Mol. Cell. Cardiol. 125, 73–86. 10.1016/j.yjmcc.2018.10.017 30342008

[B40] Goldman R. D. Gruenbaum Y. Moir R. D. Shumaker D. K. Spann T. P. (2002). Nuclear lamins: Building blocks of nuclear architecture. Genes Dev. 16, 533–547. 10.1101/gad.960502 11877373

[B41] Goldman R. D. Shumaker D. K. Erdos M. R. Eriksson M. Goldman A. E. Gordon L. B. (2004). Accumulation of mutant lamin A causes progressive changes in nuclear architecture in Hutchinson-Gilford progeria syndrome. Proc. Natl. Acad. Sci. U. S. A. 101, 8963–8968. 10.1073/pnas.0402943101 15184648 PMC428455

[B42] Gonzalez J. M. Navarro-Puche A. Casar B. Crespo P. Andres V. (2008). Fast regulation of AP-1 activity through interaction of lamin A/C, ERK1/2, and c-Fos at the nuclear envelope. J. Cell Biol. 183, 653–666. 10.1083/jcb.200805049 19015316 PMC2582892

[B43] Gotzmann J. Foisner R. (2006). A-Type lamin complexes and regenerative potential: A step towards understanding laminopathic diseases? Histochem Cell Biol. 125, 33–41. 10.1007/s00418-005-0050-8 16142451

[B44] Guilly M. N. Kolb J. P. Gosti F. Godeau F. Courvalin J. C. (1990). Lamins A and C are not expressed at early stages of human lymphocyte differentiation. Exp. Cell Res. 189, 145–147. 10.1016/0014-4827(90)90267-e 2347374

[B45] Guo D. Cheng L. Shen Y. Li W. Li Q. Zhong Y. (2020). 6-Bromoindirubin-3'-oxime (6BIO) prevents myocardium from aging by inducing autophagy. Aging (Albany NY) 12, 26047–26062. 10.18632/aging.202253 33401248 PMC7803501

[B46] Heessen S. Fornerod M. (2007). The inner nuclear envelope as a transcription factor resting place. Embo Rep. 8, 914–919. 10.1038/sj.embor.7401075 17906672 PMC2002563

[B47] Ho R. Hegele R. A. (2019). Complex effects of laminopathy mutations on nuclear structure and function. Clin. Genet. 95, 199–209. 10.1111/cge.13455 30280378

[B48] Holaska J. M. (2008). Emerin and the nuclear lamina in muscle and cardiac disease. Circ. Res. 103, 16–23. 10.1161/CIRCRESAHA.108.172197 18596264

[B49] Hutchison C. J. (2011). The role of DNA damage in laminopathy progeroid syndromes. Biochem. Soc. Trans. 39, 1715–1718. 10.1042/BST20110700 22103513

[B50] Hytiroglou P. Choi S. W. Theise N. D. Chaudhary N. Worman H. J. Thung S. N. (1993). The expression of nuclear lamins in human liver: An immunohistochemical study. Hum. Pathol. 24, 169–172. 10.1016/0046-8177(93)90296-s 8381765

[B51] Ivorra' C. Kubicek M. Gonzalez J. M. Sanz-Gonzalez S. M. Alvarez-Barrientos A. O'connor L. E. (2006). A mechanism of AP-1 suppression through interaction of c-Fos with lamin A/C. Genes & Dev. 20, 307–320. 10.1101/gad.349506 16452503 PMC1361702

[B52] Jacob K. N. Garg A. (2006). Laminopathies: Multisystem dystrophy syndromes. Mol. Genet. Metab. 87, 289–302. 10.1016/j.ymgme.2005.10.018 16364671

[B53] Jansen M. P. Machiels B. M. Hopman A. H. Broers J. L. Bot F. J. Arends J. W. (1997). Comparison of A and B-type lamin expression in reactive lymph nodes and nodular sclerosing Hodgkin's disease. Histopathology 31, 304–312. 10.1046/j.1365-2559.1997.2820881.x 9363444

[B54] Johnson B. R. Nitta R. T. Frock R. L. Mounkes L. Barbie D. A. Stewart C. L. (2004). A-type lamins regulate retinoblastoma protein function by promoting subnuclear localization and preventing proteasomal degradation. Proc. Natl. Acad. Sci. U. S. A. 101, 9677–9682. 10.1073/pnas.0403250101 15210943 PMC470734

[B55] Kang J. Y. Kang N. Yang Y. M. Hong J. H. Shin D. M. (2020). The role of Ca(2+)-NFATc1 signaling and its modulation on osteoclastogenesis. Int. J. Mol. Sci. 21, 3646. 10.3390/ijms21103646 32455661 PMC7279283

[B56] Khan Z. S. Santos J. M. Hussain F. (2018). Aggressive prostate cancer cell nuclei have reduced stiffness. Biomicrofluidics 12, 014102. 10.1063/1.5019728 29333204 PMC5750055

[B57] Kim H. J. Lee P. C. W. Hong J. H. (2022). Lamin-A/C is modulated by the involvement of histamine-mediated calcium/calmodulin-dependent kinase II in lung cancer cells. Int. J. Mol. Sci. 23, 9075. 10.3390/ijms23169075 36012358 PMC9409298

[B58] Kirkland N. J. Skalak S. H. Whitehead A. J. Hocker J. D. Beri P. Vogler G. (2023). Age-dependent Lamin changes induce cardiac dysfunction via dysregulation of cardiac transcriptional programs. Nat. Aging 3, 17–33. 10.1038/s43587-022-00323-8 36845078 PMC9956937

[B59] Klemm D. J. Leitner J. W. Watson P. Nesterova A. Reusch J. E. Goalstone M. L. (2001). Insulin-induced adipocyte differentiation. Activation of CREB rescues adipogenesis from the arrest caused by inhibition of prenylation. J. Biol. Chem. 276, 28430–28435. 10.1074/jbc.M103382200 11375992

[B60] Kong L. Schafer G. Bu H. J. Zhang Y. Zhang Y. X. Klocker H. (2012). Lamin A/C protein is overexpressed in tissue-invading prostate cancer and promotes prostate cancer cell growth, migration and invasion through the PI3K/AKT/PTEN pathway. Carcinogenesis 33, 751–759. 10.1093/carcin/bgs022 22301279

[B61] Lattanzi G. Marmiroli S. Facchini A. Maraldi N. M. (2012). Nuclear damages and oxidative stress: New perspectives for laminopathies. Eur. J. Histochem 56, e45. 10.4081/ejh.2012.e45 23361241 PMC3567764

[B62] Lazarte J. Hegele R. A. (2021). Lamin A/C missense variants: From discovery to functional validation. NPJ Genom Med. 6, 102. 10.1038/s41525-021-00266-w 34862397 PMC8642461

[B63] Le Dour C. Macquart C. Sera F. Homma S. Bonne G. Morrow J. P. (2017). Decreased WNT/β-catenin signalling contributes to the pathogenesis of dilated cardiomyopathy caused by mutations in the lamin a/C gene. Hum. Mol. Genet. 26, 333–343. 10.1093/hmg/ddw389 28069793 PMC6075603

[B64] Lee S. C. Chan J. Clement M. V. Pervaiz S. (2006). Functional proteomics of resveratrol-induced colon cancer cell apoptosis: caspase-6-mediated cleavage of lamin A is a major signaling loop. Proteomics 6, 2386–2394. 10.1002/pmic.200500366 16518869

[B65] Li W. Yeo L. S. Vidal C. Mccorquodale T. Herrmann M. Fatkin D. (2011). Decreased bone formation and osteopenia in lamin a/c-deficient mice. PLoS One 6, e19313. 10.1371/journal.pone.0019313 21547077 PMC3081846

[B66] Liddane A. G. Holaska J. M. (2021). The role of emerin in cancer progression and metastasis. Int. J. Mol. Sci. 22, 11289. 10.3390/ijms222011289 34681951 PMC8537873

[B67] Lloyd D. J. Trembath R. C. Shackleton S. (2002). A novel interaction between lamin A and SREBP1: Implications for partial lipodystrophy and other laminopathies. Hum. Mol. Genet. 11, 769–777. 10.1093/hmg/11.7.769 11929849

[B68] Lu J. T. Muchir A. Nagy P. L. Worman H. J. (2011). LMNA cardiomyopathy: Cell biology and genetics meet clinical medicine. Dis. Model Mech. 4, 562–568. 10.1242/dmm.006346 21810905 PMC3180218

[B69] Luderus M. E. Den Blaauwen J. L. De Smit O. J. Compton D. A. Van Driel R. (1994). Binding of matrix attachment regions to lamin polymers involves single-stranded regions and the minor groove. Mol. Cell Biol. 14, 6297–6305. 10.1128/mcb.14.9.6297 8065361 PMC359156

[B70] Machiels B. M. Broers J. L. Raymond Y. De Ley L. Kuijpers H. J. Caberg N. E. (1995). Abnormal A-type lamin organization in a human lung carcinoma cell line. Eur. J. Cell Biol. 67, 328–335. 8521872

[B71] Maggi L. Mavroidis M. Psarras S. Capetanaki Y. Lattanzi G. (2021). Skeletal and cardiac muscle disorders caused by mutations in genes encoding intermediate filament proteins. Int. J. Mol. Sci. 22, 4256. 10.3390/ijms22084256 33923914 PMC8073371

[B72] Malhas A. N. Vaux D. J. (2011). The nuclear envelope and its involvement in cellular stress responses. Biochem. Soc. Trans. 39, 1795–1798. 10.1042/BST20110719 22103528

[B73] Markiewicz E. Dechat T. Foisner R. Quinlan R. A. Hutchison C. J. (2002). Lamin A/C binding protein LAP2alpha is required for nuclear anchorage of retinoblastoma protein. Mol. Biol. Cell 13, 4401–4413. 10.1091/mbc.e02-07-0450 12475961 PMC138642

[B74] Marmiroli S. Bertacchini J. Beretti F. Cenni V. Guida M. De Pol A. (2009). A-Type lamins and signaling: The PI 3-kinase/Akt pathway moves forward. J. Cell Physiol. 220, 553–561. 10.1002/jcp.21807 19479937

[B75] Matias I. Diniz L. P. Damico I. V. Araujo A. P. B. Neves L. D. S. Vargas G. (2022). Loss of lamin-B1 and defective nuclear morphology are hallmarks of astrocyte senescence *in vitro* and in the aging human hippocampus. Aging Cell 21, e13521. 10.1111/acel.13521 34894056 PMC8761005

[B76] Mckenna T. Rosengardten Y. Viceconte N. Baek J. H. Grochova D. Eriksson M. (2014). Embryonic expression of the common progeroid lamin A splice mutation arrests postnatal skin development. Aging Cell 13, 292–302. 10.1111/acel.12173 24305605 PMC4331787

[B77] Merideth M. A. Gordon L. B. Clauss S. Sachdev V. Smith A. C. Perry M. B. (2008). Phenotype and course of Hutchinson-Gilford progeria syndrome. N. Engl. J. Med. 358, 592–604. 10.1056/NEJMoa0706898 18256394 PMC2940940

[B78] Mislow J. M. Holaska J. M. Kim M. S. Lee K. K. Segura-Totten M. Wilson K. L. (2002). Nesprin-1alpha self-associates and binds directly to emerin and lamin A *in vitro* . FEBS Lett. 525, 135–140. 10.1016/s0014-5793(02)03105-8 12163176

[B79] Moiseeva O. Lessard F. Acevedo-Aquino M. Vernier M. Tsantrizos Y. S. Ferbeyre G. (2015). Mutant lamin A links prophase to a p53 independent senescence program. Cell Cycle 14, 2408–2421. 10.1080/15384101.2015.1053671 26029982 PMC4614274

[B80] Moss S. F. Krivosheyev V. De Souza A. Chin K. Gaetz H. P. Chaudhary N. (1999). Decreased and aberrant nuclear lamin expression in gastrointestinal tract neoplasms. Gut 45, 723–729. 10.1136/gut.45.5.723 10517909 PMC1727735

[B81] Mounkes L. C. Kozlov S. Hernandez L. Sullivan T. Stewart C. L. (2003). A progeroid syndrome in mice is caused by defects in A-type lamins. Nature 423, 298–301. 10.1038/nature01631 12748643

[B82] Mounkes L. C. Kozlov S. V. Rottman J. N. Stewart C. L. (2005). Expression of an LMNA-N195K variant of A-type lamins results in cardiac conduction defects and death in mice. Hum. Mol. Genet. 14, 2167–2180. 10.1093/hmg/ddi221 15972724

[B83] Muchir A. Pavlidis P. Decostre V. Herron A. J. Arimura T. Bonne G. (2007). Activation of MAPK pathways links LMNA mutations to cardiomyopathy in Emery-Dreifuss muscular dystrophy. J. Clin. Invest. 117, 1282–1293. 10.1172/JCI29042 17446932 PMC1849984

[B84] Muchir A. Van Engelen B. G. Lammens M. Mislow J. M. Mcnally E. Schwartz K. (2003). Nuclear envelope alterations in fibroblasts from LGMD1B patients carrying nonsense Y259X heterozygous or homozygous mutation in lamin A/C gene. Exp. Cell Res. 291, 352–362. 10.1016/j.yexcr.2003.07.002 14644157

[B85] Muchir A. Wu W. Choi J. C. Iwata S. Morrow J. Homma S. (2012). Abnormal p38α mitogen-activated protein kinase signaling in dilated cardiomyopathy caused by lamin A/C gene mutation. Hum. Mol. Genet. 21, 4325–4333. 10.1093/hmg/dds265 22773734 PMC3441127

[B86] Naeem A. S. Zhu Y. Di W. L. Marmiroli S. O'shaughnessy R. F. L. (2015). AKT1-mediated Lamin A/C degradation is required for nuclear degradation and normal epidermal terminal differentiation. Cell Death Differ. 22, 2123–2132. 10.1038/cdd.2015.62 26045045 PMC4816115

[B87] Nakamura M. Yamada M. Ohsawa T. Morisawa H. Nishine T. Nishimura O. (2006). Phosphoproteomic profiling of human SH-SY5Y neuroblastoma cells during response to 6-hydroxydopamine-induced oxidative stress. Biochim. Biophys. Acta 1763, 977–989. 10.1016/j.bbamcr.2006.07.008 16949164

[B88] Olaopa M. A. Ai T. Chao B. Xiao X. S. Vatta M. Habecker B. A. (2021). Phosphorylation of Lamin A/C at serine 22 modulates Na(v)1.5 function. Physiol. Rep. 9, e15121. 10.14814/phy2.15121 34806324 PMC8606869

[B89] Osorio F. G. Barcena C. Soria-Valles C. Ramsay A. J. De Carlos F. Cobo J. (2012). Nuclear lamina defects cause ATM-dependent NF-κB activation and link accelerated aging to a systemic inflammatory response. Genes Dev. 26, 2311–2324. 10.1101/gad.197954.112 23019125 PMC3475803

[B90] Ovsiannikova N. L. Lavrushkina S. V. Ivanova A. V. Mazina L. M. Zhironkina O. A. Kireev I. I. (2021). Lamin A as a determinant of mechanical properties of the cell nucleus in health and disease. Biochemistry-Moscow 86, 1288–1300. 10.1134/S0006297921100102 34903160

[B91] Parnaik V. K. (2008). Role of nuclear lamins in nuclear organization, cellular signaling, and inherited diseases. Int. Rev. Cell Mol. Biol. 266, 157–206. 10.1016/S1937-6448(07)66004-3 18544494

[B92] Patil S. Sengupta K. (2021). Role of A- and B-type lamins in nuclear structure-function relationships. Biol. Cell 113, 295–310. 10.1111/boc.202000160 33638183

[B93] Paulin-Levasseur M. Scherbarth A. Traub U. Traub P. (1988). Lack of lamins A and C in mammalian hemopoietic cell lines devoid of intermediate filament proteins. Eur. J. Cell Biol. 47, 121–131. 3068054

[B94] Pekovic V. Gibbs-Seymour I. Markiewicz E. Alzoghaibi F. Benham A. M. Edwards R. (2011). Conserved cysteine residues in the mammalian lamin A tail are essential for cellular responses to ROS generation. Aging Cell 10, 1067–1079. 10.1111/j.1474-9726.2011.00750.x 21951640

[B95] Pekovic V. Harborth J. Broers J. L. Ramaekers F. C. Van Engelen B. Lammens M. (2007). Nucleoplasmic LAP2alpha-lamin A complexes are required to maintain a proliferative state in human fibroblasts. J. Cell Biol. 176, 163–172. 10.1083/jcb.200606139 17227891 PMC2063936

[B96] Piekarowicz K. Machowska M. Dratkiewicz E. Lorek D. Madej-Pilarczyk A. Rzepecki R. (2017). The effect of the lamin A and its mutants on nuclear structure, cell proliferation, protein stability, and mobility in embryonic cells. Chromosoma 126, 501–517. 10.1007/s00412-016-0610-9 27534416 PMC5509783

[B97] Pradhan R. Nallappa M. J. Sengupta K. (2020). Lamin A/C modulates spatial organization and function of the Hsp70 gene locus via nuclear myosin I. J. Cell Sci. 133, jcs236265. 10.1242/jcs.236265 31988151

[B98] Prokocimer M. Davidovich M. Nissim-Rafinia M. Wiesel-Motiuk N. Bar D. Z. Barkan R. (2009). Nuclear lamins: Key regulators of nuclear structure and activities. J. Cell Mol. Med. 13, 1059–1085. 10.1111/j.1582-4934.2008.00676.x 19210577 PMC4496104

[B99] Qi Y. X. Yao Q. P. Huang K. Shi Q. Zhang P. Wang G. L. (2016). Nuclear envelope proteins modulate proliferation of vascular smooth muscle cells during cyclic stretch application. Proc. Natl. Acad. Sci. U. S. A. 113, 5293–5298. 10.1073/pnas.1604569113 27114541 PMC4868428

[B100] Rao L. Perez D. White E. (1996). Lamin proteolysis facilitates nuclear events during apoptosis. J. Cell Biol. 135, 1441–1455. 10.1083/jcb.135.6.1441 8978814 PMC2133948

[B101] Rauner M. Sipos W. Goettsch C. Wutzl A. Foisner R. Pietschmann P. (2009). Inhibition of lamin A/C attenuates osteoblast differentiation and enhances RANKL-dependent osteoclastogenesis. J. Bone Min. Res. 24, 78–86. 10.1359/jbmr.080902 18767923

[B102] Raz V. Vermolen B. J. Garini Y. Onderwater J. J. Mommaas-Kienhuis M. A. Koster A. J. (2008). The nuclear lamina promotes telomere aggregation and centromere peripheral localization during senescence of human mesenchymal stem cells. J. Cell Sci. 121, 4018–4028. 10.1242/jcs.034876 19056671

[B103] Ricci A. Orazi S. Biancucci F. Magnani M. Menotta M. (2021). The nucleoplasmic interactions among Lamin A/C-pRB-LAP2α-E2F1 are modulated by dexamethasone. Sci. Rep. 11, 10099. 10.1038/s41598-021-89608-3 33980953 PMC8115688

[B104] Rivas D. Akter R. Duque G. (2007). Inhibition of protein farnesylation arrests adipogenesis and affects PPARgamma expression and activation in differentiating mesenchymal stem cells. PPAR Res. 2007, 81654. 10.1155/2007/81654 18274630 PMC2220071

[B105] Rogerson C. Wotherspoon D. J. Tommasi C. Button R. W. O'shaughnessy R. F. L. (2021). Akt1-associated actomyosin remodelling is required for nuclear lamina dispersal and nuclear shrinkage in epidermal terminal differentiation. Cell Death Differ. 28, 1849–1864. 10.1038/s41418-020-00712-9 33462407 PMC8184862

[B106] Sagelius H. Rosengardten Y. Hanif M. Erdos M. R. Rozell B. Collins F. S. (2008). Targeted transgenic expression of the mutation causing Hutchinson-Gilford progeria syndrome leads to proliferative and degenerative epidermal disease. J. Cell Sci. 121, 969–978. 10.1242/jcs.022913 18334552

[B107] Saha B. Zitnik G. Johnson S. Nguyen Q. Risques R. A. Martin G. M. (2013). DNA damage accumulation and TRF2 degradation in atypical Werner syndrome fibroblasts with LMNA mutations. Front. Genet. 4, 129. 10.3389/fgene.2013.00129 23847654 PMC3701863

[B108] Sakurai K. Sugiura H. Matsumoto M. Uehara M. (2002). Occurrence of patchy parakeratosis in normal-appearing skin in patients with active atopic dermatitis and in patients with healed atopic dermatitis: A cause of impaired barrier function of the atopic skin. J. Dermatol Sci. 30, 37–42. 10.1016/s0923-1811(02)00047-6 12354418

[B109] Sengupta D. Ali S. N. Bhattacharya A. Mustafi J. Mukhopadhyay A. Sengupta K. (2022). A deep hybrid learning pipeline for accurate diagnosis of ovarian cancer based on nuclear morphology. PLoS One 17, e0261181. 10.1371/journal.pone.0261181 34995293 PMC8741040

[B110] Sieprath T. Darwiche R. De Vos W. H. (2012). Lamins as mediators of oxidative stress. Biochem. Biophysical Res. Commun. 421, 635–639. 10.1016/j.bbrc.2012.04.058 22538370

[B111] Smith E. D. Garza-Gongora A. G. Macquarrie K. L. Kosak S. T. (2018). Interstitial telomeric loops and implications of the interaction between TRF2 and lamin A/C. Differentiation 102, 19–26. 10.1016/j.diff.2018.06.001 29979997

[B112] Stadelmann B. Khandjian E. Hirt A. Luthy A. Weil R. Wagner H. P. (1990). Repression of nuclear lamin A and C gene expression in human acute lymphoblastic leukemia and non-Hodgkin's lymphoma cells. Leuk. Res. 14, 815–821. 10.1016/0145-2126(90)90076-l 2232854

[B113] Steinle N. I. Kazlauskaite R. Imumorin I. G. Hsueh W. C. Pollin T. I. O'connell J. R. (2004). Variation in the lamin A/C gene: Associations with metabolic syndrome. Arterioscler. Thromb. Vasc. Biol. 24, 1708–1713. 10.1161/01.ATV.0000136384.53705.c9 15205219

[B114] Sullivan T. Escalante-Alcalde D. Bhatt H. Anver M. Bhat N. Nagashima K. (1999). Loss of A-type lamin expression compromises nuclear envelope integrity leading to muscular dystrophy. J. Cell Biol. 147, 913–920. 10.1083/jcb.147.5.913 10579712 PMC2169344

[B115] Sur-Erdem I. Hussain M. S. Asif M. Pinarbasi N. Aksu A. C. Noegel A. A. (2020). Nesprin-1 impact on tumorigenic cell phenotypes. Mol. Biol. Rep. 47, 921–934. 10.1007/s11033-019-05184-w 31741263

[B116] Swift J. Ivanovska I. L. Buxboim A. Harada T. Dingal P. C. Pinter J. (2013). Nuclear lamin-A scales with tissue stiffness and enhances matrix-directed differentiation. Science 341, 1240104. 10.1126/science.1240104 23990565 PMC3976548

[B117] Sylvius N. Tesson F. (2006). Lamin A/C and cardiac diseases. Curr. Opin. Cardiol. 21, 159–165. 10.1097/01.hco.0000221575.33501.58 16601451

[B118] Takahashi A. Alnemri E. S. Lazebnik Y. A. Fernandesalnemri T. Litwack G. Moir R. D. (1996). Cleavage of lamin A by Mch2 alpha but not CPP32: Multiple interleukin 1 beta-converting enzyme-related proteases with distinct substrate recognition properties are active in apoptosis. Proc. Natl. Acad. Sci. U. S. A. 93, 8395–8400. 10.1073/pnas.93.16.8395 8710882 PMC38682

[B119] Tong J. Li W. Vidal C. Yeo L. S. Fatkin D. Duque G. (2011). Lamin A/C deficiency is associated with fat infiltration of muscle and bone. Mech. Ageing Dev. 132, 552–559. 10.1016/j.mad.2011.09.004 21982926

[B120] Tran J. R. Chen H. Y. Zheng X. B. Zheng Y. X. (2016). Lamin in inflammation and aging. Curr. Opin. Cell Biol. 40, 124–130. 10.1016/j.ceb.2016.03.004 27023494 PMC4887417

[B121] Travina A. O. Ilicheva N. V. Mittenberg A. G. Shabelnikov S. V. Kotova A. V. Podgornaya O. I. (2021). The long linker region of telomere-binding protein TRF2 is responsible for interactions with lamins. Int. J. Mol. Sci. 22, 3293. 10.3390/ijms22073293 33804854 PMC8036907

[B122] Urciuoli E. D'oria V. Petrini S. Peruzzi B. (2021). Lamin A/C mechanosensor drives tumor cell aggressiveness and adhesion on substrates with tissue-specific elasticity. Front. Cell Dev. Biol. 9, 712377. 10.3389/fcell.2021.712377 34595168 PMC8476891

[B123] Vadrot N. Duband-Goulet I. Cabet E. Attanda W. Barateau A. Vicart P. (2015). The p.R482W substitution in A-type lamins deregulates SREBP1 activity in Dunnigan-type familial partial lipodystrophy. Hum. Mol. Genet. 24, 2096–2109. 10.1093/hmg/ddu728 25524705

[B124] Van Berlo J. H. Voncken J. W. Kubben N. Broers J. L. Duisters R. Van Leeuwen R. E. (2005). A-type lamins are essential for TGF-beta1 induced PP2A to dephosphorylate transcription factors. Hum. Mol. Genet. 14, 2839–2849. 10.1093/hmg/ddi316 16115815

[B125] Vaughan O. A. Alvarez-Reyes M. Bridger J. M. Broers J. L. V. Ramaekers F. C. S. Wehnert M. (2001). Both emerin and lamin C depend on lamin A for localization at the nuclear envelope. J. Cell Sci. 114, 2577–2590. 10.1242/jcs.114.14.2577 11683386

[B126] Vidal C. Bermeo S. Fatkin D. Duque G. (2012). Role of the nuclear envelope in the pathogenesis of age-related bone loss and osteoporosis. Bonekey Rep. 1, 62. 10.1038/bonekey.2012.62 23951459 PMC3727739

[B127] Virtanen L. Holm E. Halme M. West G. Lindholm F. Gullmets J. (2023). Lamin A/C phosphorylation at serine 22 is a conserved heat shock response to regulate nuclear adaptation during stress. J. Cell Sci. 136, jcs259788. 10.1242/jcs.259788 36695453 PMC10022683

[B128] Wang Y. Dobreva G. (2023). Epigenetics in LMNA-related cardiomyopathy. Cells 12, 783. 10.3390/cells12050783 36899919 PMC10001118

[B129] Wang Y. Herron A. J. Worman H. J. (2006). Pathology and nuclear abnormalities in hearts of transgenic mice expressing M371K lamin A encoded by an LMNA mutation causing Emery-Dreifuss muscular dystrophy. Hum. Mol. Genet. 15, 2479–2489. 10.1093/hmg/ddl170 16825283

[B130] Wang Y. Jiang J. He L. Gong G. Wu X. (2019). Effect of lamin-A expression on migration and nuclear stability of ovarian cancer cells. Gynecol. Oncol. 152, 166–176. 10.1016/j.ygyno.2018.10.030 30384980

[B131] Willis N. D. Cox T. R. Rahman-Casans S. F. Smits K. Przyborski S. A. Van Den Brandt P. (2008). Lamin A/C is a risk biomarker in colorectal cancer. Plos One 3, e2988. 10.1371/journal.pone.0002988 18714339 PMC2496895

[B132] Worman H. J. (2012). Nuclear lamins and laminopathies. J. Pathol. 226, 316–325. 10.1002/path.2999 21953297 PMC6673656

[B133] Worman H. J. Schirmer E. C. (2015). Nuclear membrane diversity: Underlying tissue-specific pathologies in disease? Curr. Opin. Cell Biol. 34, 101–112. 10.1016/j.ceb.2015.06.003 26115475 PMC4522394

[B134] Wu Z. Wu L. Weng D. Xu D. Geng J. Zhao F. (2009). Reduced expression of lamin A/C correlates with poor histological differentiation and prognosis in primary gastric carcinoma. J. Exp. Clin. Cancer Res. 28, 8. 10.1186/1756-9966-28-8 19144202 PMC2632624

[B135] Yang S. H. Bergo M. O. Toth J. I. Qiao X. Hu Y. Sandoval S. (2005). Blocking protein farnesyltransferase improves nuclear blebbing in mouse fibroblasts with a targeted Hutchinson-Gilford progeria syndrome mutation. Proc. Natl. Acad. Sci. U. S. A. 102, 10291–10296. 10.1073/pnas.0504641102 16014412 PMC1174929

[B136] Yang S. H. Qiao X. Fong L. G. Young S. G. (2008). Treatment with a farnesyltransferase inhibitor improves survival in mice with a Hutchinson-Gilford progeria syndrome mutation. Biochim. Biophys. Acta 1781, 36–39. 10.1016/j.bbalip.2007.11.003 18082640 PMC2266774

[B137] Yasuda H. Shima N. Nakagawa N. Yamaguchi K. Kinosaki M. Mochizuki S. (1998). Osteoclast differentiation factor is a ligand for osteoprotegerin osteoclastogenesis-inhibitory factor and is identical to TRANCE/RANKL. Proc. Natl. Acad. Sci. U. S. A. 95, 3597–3602. 10.1073/pnas.95.7.3597 9520411 PMC19881

[B138] Zastrow M. S. Vlcek S. Wilson K. L. (2004). Proteins that bind A-type lamins: Integrating isolated clues. J. Cell Sci. 117, 979–987. 10.1242/jcs.01102 14996929

[B139] Zhang X. Lv Y. (2017). Suspension state increases reattachment of breast cancer cells by up-regulating lamin A/C. Biochim. Biophys. Acta Mol. Cell Res. 1864, 2272–2282. 10.1016/j.bbamcr.2017.09.006 28919351

[B140] Zhang Y. Wang J. Huang W. Cai J. Ba J. Wang Y. (2018). Nuclear Nestin deficiency drives tumor senescence via lamin A/C-dependent nuclear deformation. Nat. Commun. 9, 3613. 10.1038/s41467-018-05808-y 30190500 PMC6127343

[B141] Zhao B. Y. Tang M. Lv Y. G. (2022). Shear stress regulates the migration of suspended breast cancer cells by nuclear lamina protein A/C and large tumor suppressor through yes-associated protein. Hum. Cell 35, 583–598. 10.1007/s13577-021-00666-1 34984662

